# Ninety percent circular polarization detected in a repeating fast radio burst

**DOI:** 10.1093/nsr/nwae293

**Published:** 2024-09-09

**Authors:** Jinchen Jiang, Jiangwei Xu, Jiarui Niu, Kejia Lee, Weiwei Zhu, Bing Zhang, Yuanhong Qu, Heng Xu, Dejiang Zhou, Shunshun Cao, Weiyang Wang, Bojun Wang, Shuo Cao, Yongkun Zhang, Chunfeng Zhang, Hengqian Gan, Jinlin Han, Longfei Hao, Yuxiang Huang, Peng Jiang, Dongzi Li, Hui Li, Ye Li, Zhixuan Li, Rui Luo, Yunpeng Men, Lei Qian, Jinghai Sun, Lin Wang, Yonghua Xu, Renxin Xu, Yuanpei Yang, Rui Yao, Youling Yue, Dongjun Yu, Jianping Yuan, Yan Zhu

**Affiliations:** National Astronomical Observatories, Chinese Academy of Sciences (CAS), Beijing 100101, China; Department of Astronomy, Peking University, Beijing 100871, China; Kavli Institute for Astronomy and Astrophysics, Peking University, Beijing 100871, China; National Astronomical Observatories, Chinese Academy of Sciences (CAS), Beijing 100101, China; National Astronomical Observatories, Chinese Academy of Sciences (CAS), Beijing 100101, China; Department of Astronomy, Peking University, Beijing 100871, China; Beijing Laser Acceleration Innovation Center, Peking University, Beijing 101400, China; National Astronomical Observatories, Chinese Academy of Sciences (CAS), Beijing 100101, China; Nevada Center for Astrophysics, University of Nevada, Las Vegas 89154, USA; Department of Physics and Astronomy, University of Nevada, Las Vegas 89154, USA; Nevada Center for Astrophysics, University of Nevada, Las Vegas 89154, USA; Department of Physics and Astronomy, University of Nevada, Las Vegas 89154, USA; National Astronomical Observatories, Chinese Academy of Sciences (CAS), Beijing 100101, China; National Astronomical Observatories, Chinese Academy of Sciences (CAS), Beijing 100101, China; Department of Astronomy, Peking University, Beijing 100871, China; School of Astronomy and Space Science, University of Chinese Academy of Sciences, Beijing 100049, China; National Astronomical Observatories, Chinese Academy of Sciences (CAS), Beijing 100101, China; Yunnan Observatories, CAS, Kunming 650216, China; National Astronomical Observatories, Chinese Academy of Sciences (CAS), Beijing 100101, China; National Astronomical Observatories, Chinese Academy of Sciences (CAS), Beijing 100101, China; National Astronomical Observatories, Chinese Academy of Sciences (CAS), Beijing 100101, China; National Astronomical Observatories, Chinese Academy of Sciences (CAS), Beijing 100101, China; Yunnan Observatories, CAS, Kunming 650216, China; Yunnan Observatories, CAS, Kunming 650216, China; National Astronomical Observatories, Chinese Academy of Sciences (CAS), Beijing 100101, China; Department of Astrophysical Sciences, Princeton University, Princeton 08544, USA; National Astronomical Observatories, Chinese Academy of Sciences (CAS), Beijing 100101, China; Purple Mountain Observatory, CAS, Nanjing 210008, China; Yunnan Observatories, CAS, Kunming 650216, China; School of Physics and Electronic Engineering, Guangzhou University, Guangzhou 510006, China; Electronic Department, Max-Planck institut für Radioastronomie, Bonn 53121, Germany; National Astronomical Observatories, Chinese Academy of Sciences (CAS), Beijing 100101, China; National Astronomical Observatories, Chinese Academy of Sciences (CAS), Beijing 100101, China; Kavli Institute for Astronomy and Astrophysics, Peking University, Beijing 100871, China; Yunnan Observatories, CAS, Kunming 650216, China; Department of Astronomy, Peking University, Beijing 100871, China; State Key Laboratory of Nuclear Physics and Technology, Peking University, Beijing 100871, China; South-Western Institute For Astronomy Research, Yunnan University, Kunming 650504, China; National Astronomical Observatories, Chinese Academy of Sciences (CAS), Beijing 100101, China; National Astronomical Observatories, Chinese Academy of Sciences (CAS), Beijing 100101, China; National Astronomical Observatories, Chinese Academy of Sciences (CAS), Beijing 100101, China; Xinjiang Astronomical Observatory, CAS, Urumqi 830011, China; National Astronomical Observatories, Chinese Academy of Sciences (CAS), Beijing 100101, China

**Keywords:** radio astronomy, fast radio burst, polarization

## Abstract

Fast radio bursts (FRBs) are extra-galactic sources with unknown physical mechanisms. They emit millisecond-duration radio pulses with isotropic equivalent energy of $10^{36}$–$10^{41}$ ergs. This corresponds to a brightness temperature of FRB emission typically reaching the level of $10^{36}$ K, but can be as high as above $10^{40}$ K for sub-microsecond timescale structures, suggesting the presence of underlying coherent relativistic radiation mechanisms. Polarization carries key information to understand the physical origin of FRBs, with linear polarization usually tracing the geometric configuration of magnetic fields and circular polarization probing both intrinsic radiation mechanisms and propagation effects. Here we show that the repeating source FRB 20201124A emits $90.9\%\pm 1.1\%$ circularly polarized radio pulses. Such a high degree of circular polarization was unexpected in theory and unprecedented in observation in the case of FRBs, since such a high degree of circular polarization was only common among solar or Jovian radio activities, attributed to the sub-relativistic electrons. We note that there is no obvious correlation between the degree of circular polarization and burst fluence. Besides the high degree of circular polarization, we also detected a rapid swing and orthogonal jump in the position angle of linear polarization. The detection of high-degree circular polarization in FRB 20201124A, together with its linear polarization properties that show orthogonal modes, place strong constraints on FRB physical mechanisms, calling for an interplay between magnetospheric radiation and propagation effects in shaping the observed FRB radiation.

## Teaser

The largest radio telescope FAST detects 90% circular polarization and rapidly changing linear polarization in the bursts of a fast radio burst (FRB) repeater, imposing new constraint on radiative mechanism of FRBs.

## INTRODUCTION

Within the framework of the magnetar engine as suggested by the detection of the Galactic FRB 20200428D [1,2], two major types of models have been proposed [3]: the far-away, gamma-ray-burst- (GRB) like models that invoke a synchrotron maser in highly magnetized, relativistic shocks [4,5] and the closer-in, pulsar-like models that invoke radio emissions from magnetospheres [6–11]. Previous observations already provided crucial polarization properties of FRBs, including linear polarization angle swings [12–19], and oscillations between linear and circular polarizations [14].

Moderate levels of circular polarization from 5% to 57% have been reported for FRBs without observed repetition, i.e. for FRB 20110523A [20], 20140514A [21], 20160102A [22], 20180309A [23], 20180311A [23], 20180714A [23], 20180924B [24], 20181112A [25,26], 20190102C [24], 20190608B [24], 20190611B [24] and 20191219F [27]. Repeating FRBs mostly show a low level of circular polarization (a few percent) and only a few of them

emit significant circular polarization for a small fraction of bursts, i.e. FRB 20190520B [28] (42%), FRB 20201124A [14–18] ($\sim\! 75\%$), FRB 20121102A [29] (64%) and FRB 20220912A [19,30,31] ($70\%$). Among the sources, FRB 20201124A is an active repeating FRB source with an event rate as high as 542 hr$^{-1}$ during its active window [14,17,32–34]. Previous observations have detected 1863 bursts from the source using the five-hundred-meter aperture spherical radio telescope (FAST) in the L-band (centered at 1.25 GHz) during one of its active periods between March and May 2021 [14]. It entered a new period of activity on 21 September 2021 according to the alert from the Canadian Hydrogen Intensity Mapping Experiment (CHIME) [35]. Following the detections, we scheduled daily FAST observations to monitor the source between 25 September and 2 October 2021 [17,32–34]. Here we report the detection of highly circularly polarized (with the highest degree of circular polarization being $90.9\%\pm 1.1\%$) radio pulses from FRB 20201124A with FAST. The observation serves as a touchstone for FRB radiation models and suggests extreme conditions for the generation and propagation of FRBs in the nearby environment.

## RESULTS

**Figure 1.**
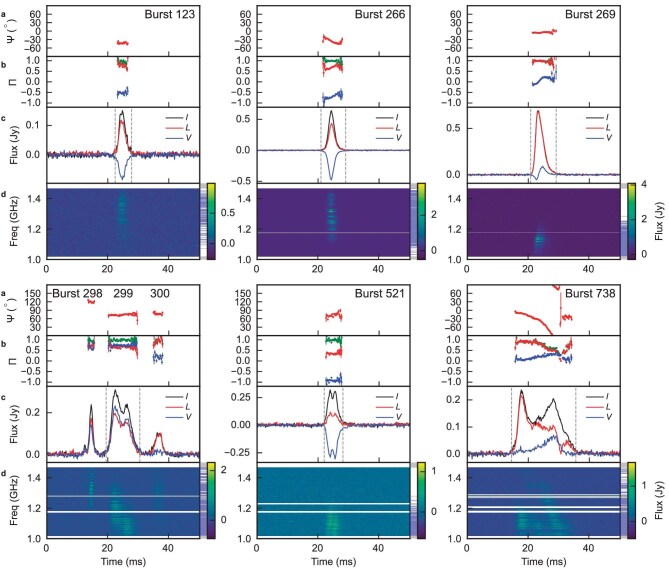
Polarimetric results of a selected sample of bursts with high degrees of circular polarization or abrupt jumps in the linear polarization position angle. For each burst, we plot (a) the position angle, (b) the degree of linear (red), circular (blue) and total polarization (green) as a function of burst time, (c) the burst profile of total intensity (black), linear polarization (red) and circular polarization (blue) and (d) dynamic spectra of the bursts, i.e. intensity as a function of time and frequency. On the right side of each spectrum, the gray shades denote the removed frequency channels affected by radio frequency interference and the 20-MHz band edges, and the blue shades denote the frequency channels where the burst signal appears. The color bar manifests the total intensity in the spectrum. Here, the frequency resolution of spectra is 1.95 MHz per channel, and the time resolution of the light curves and spectra is 196 $\mu$s per sample. Error bars are at the 68% confidence level. Bursts are de-dispersed with the daily average dispersion measure values published previously [32], which are $412.5\,\mathrm{pc\, cm^{-3}}$ for Bursts 123, 266 and 269, and $411.6\,\mathrm{pc\, cm^{-3}}$ for other bursts in this figure. Bursts 298, 299 and 300 are plotted in the same panel as they are very close in time.

In the new active window, we detected a group of highly circularly polarized bursts from FRB 20201124A and found evidence of orthogonal polarization modes, i.e. dual linear polarization modes with orthogonal directions. In a total of 536 bursts with a signal-to-noise ratio ($\mathrm{S/N}$) higher than 50 out of more than 800 bursts above the detection threshold $\mathrm{S/N}\ge 7$, we detected a group of 15 bursts with average degrees of circular polarization $\Pi _{\rm v}\equiv |V|/I> 50\%$ and 106 bursts with $\Pi _{\rm v} \ge 20\%$. A selected sample of bursts with significant circular polarization is shown in Fig. 1, while other bursts with peak $\Pi _{\rm v}> 50\%$ are shown in Figs S4–S7 within the online Supplementary data and their properties are summarized in Table S1.

As shown in Fig. 1, the time averaged $\Pi _{\rm v}$ of Bursts 266, 299 and 521 were $-71.1\%\pm 0.4\%$, $73.5\%\pm 0.7\%$ and $-90.9\%\pm 1.1\%$, respectively, while the time resolved $\Pi _{\rm v}$ could be even higher. The degree of circular polarization of Burst 521 is larger than any FRB bursts previously reported. The two bursts have opposite handedness of circular polarization. In Burst 299, the circular polarization is left handed, while the right-hand mode dominates Burst 521. The burst profiles of both bursts showed a double-peak structure in total intensity $I$, linear polarization intensity $L$ and circular polarization intensity $V$. The linear polarization position angle (PA) remained constant across the two peaks.

In Fig. 1, we also show the two adjacent bursts (Bursts 298 and 300) of Burst 299, as they are only separated by a few tens of milliseconds from Burst 299. Despite the temporal proximity, these three bursts show rather different polarization properties. The degree of circular polarization of Burst 298 is similar to that of Burst 299. However, the PA changed abruptly by $\approx 60^\circ$. Bursts 299 and 300 had the same PA, while the degree of circular polarization dropped from $73.5\%$ to $12.2\%\pm 1.9\%$ within less than 20 ms. In the spectral domain, the degree of linear polarization ($\Pi _{\rm L}\equiv L/I$) and $\Pi _{\rm v}$ remained almost constant across the observed bandwidth of bursts (Fig. S1), i.e. there is no obvious polarization evolution or oscillations [14] from 1.0 to 1.4 GHz.

Despite the similarities in Bursts 299 and 521, the circular polarization phenomena for FRB 20201124A have great diversity. From the morphology perspective, not all the high degree of circular polarization is associated with the double-peak pulse structure like Bursts 299 and 521. As an example, Burst 123 in Fig. 1 with $\Pi _{\rm V}=-52\%$ has a single peak pulse. One can also note clear variations of $\Pi _{\rm v}$ across the phase in Burst 123, opposite to the case of Bursts 299 and 521. For the bursts with lower degrees of circular polarization, we have found single-peak pulses with circular polarization sign reversal (Burst 269), which is shown in Fig. 1. We also identified abrupt $90^\circ$ jumps in the PA of linear polarization. An example (Burst 738) is shown in Fig. 1, where a $90^\circ$ jump occurs at 30 ms. One can note that the degree of circular polarization peaked at the time of the 90$^\circ$ PA jump.

## DISCUSSION

The substantially high circular polarization degree observed in a few bursts provides valuable insights into the coherent radiation mechanism of FRBs. Our immediate conclusion is that the far-way GRB-like models, which invoke maser-type emission from relativistic shocks outside the magnetosphere [4,5], are not supported by the data. In general, circular polarization of emission can be generated either from intrinsic radiation mechanisms or through propagation effects. Both mechanisms fail to accommodate the data for GRB-like models. First, it has been shown [11] that the 90% circular polarization from a bright burst cannot be generated from the intrinsic synchrotron maser (the relativistic version of the cyclotron maser [36]) model. This is because the synchrotron maser model requires ordered magnetic fields in the shock plane, and the observed radiation predominately carries high linear polarization. Circular polarization is possible with off-beam viewing geometry, but these high-$\Pi _{\rm v}$ bursts are expected to have much lower fluxes than other bursts, inconsistent with the bright high-$\Pi _{\rm v}$ bursts we detected. GRB-like models are also disfavored due to rapid swings in the PA curves, which have been observed in the emissions of repeaters FRB 20180301A [13] and FRB 20220912A [19].

Our results also challenge the out-of-magnetosphere propagation models for the circular polarization. Propagation models might plausibly incorporate Faraday conversion and synchrotron or cyclotron absorption to generate a high degree of circular polarization [11,14,37–40]. However, these models encounter difficulties in elucidating the dynamics observed in the three successive bursts within 30 ms, Bursts 298, 299 and 300, as depicted in Fig. 1. As out-of-magnetosphere propagation effects will simultaneously affect both linear and circular polarizations for all pulses, intricate conditions have to be satisfied, such that (1) the PA changed significantly while $\Pi _{\rm v}$ and $\Pi _{\rm L}$ remained nearly constant (Bursts 298 and 299), and (2) $\Pi _{\rm v}$ changed while the PA remained constant (Bursts 299 and 300). It is essentially impossible for an external plasma to make such abrupt changes within a timescale of 30 ms. Furthermore, Bursts 521 and 123 exhibited frequency-independent degrees of circular or linear polarization, rendering the models centered solely on propagation effects increasingly difficult, because both Faraday conversion and synchrotron/cyclotron absorption processes will typically exhibit frequency-dependent polarization. Furthermore, the absorption-related models require a non-negligible value of the optical depth to produce high circular polarization [11,41], and thus predict a lower flux for high-$\Pi _{\rm v}$ bursts. As a result, these mechanisms also suffer from the flux problem similar to that the synchrotron maser emission model faces.

The data are more consistent with pulsar-like models that invoke emission inside a magnetar magnetosphere. For the intrinsic radiation models, two widely discussed magnetospheric mechanisms to explain the high brightness temperatures of FRB radio emission [42] involve either curvature radiation [6–8] or inverse Compton scattering (ICS) mechanisms [10,43,44] of particle bunches. Regarding curvature radiation [9], circular polarization can be generated, when the observer’s line of sight slightly deviates from the center of the radiation beam, resulting in seeing the circular motion of electrons and detecting the corresponding circular polarization. For ICS, the scattered emission by a single particle is linearly polarized, but circular polarization can be generated as a consequence of emission from a bunch with a certain geometry. In particular, when the line of sight misaligns with the direction of the ICS electron bunch, circular polarization could be detected, as different phases are superposed to the scattered radiation induced by the different parts of the bunch [11]. A high circular polarization degree can be generated slightly off-axis, even when the line of sight is still within the $1/\gtrsimmma$ relativistic beaming cone of the relativistic bunch (γ being the Lorentz factor of the bunch).

We noted the sign reversal of $\Pi _{\rm v}$ with an example showing in Burst 269. This phenomenon closely aligns with the predictions of both curvature radiation and ICS models, which predict the reversal of the sign of circular polarization when the line of sight sweeps across the meridian plane of the electron’s trajectory, and, hence, provides strong support to the magnetospheric origin of FRB emission. In the mean time, our observations also provide constraints on these models. In general, since circular polarization is observed off-beam in both models, one should generally expect a systematically fainter population for the high-$\Pi _{\rm v}$ bursts. We compared the fluence distributions of bursts with $\Pi _{\rm v} \ge 50\%$ and $\Pi _{\rm v} \le 50\%$ using the two-sample Kolmogorov–Smirnov test and could not find evidence that the bursts with $\Pi _{\rm v}\ge 50\%$ are systematically fainter (see Section S4). Our simulations showed that one would detect the difference at a confidence level higher than 95%, if the energies of $\Pi _{\rm v}\ge 50\%$ bursts are reduced to 65% of the observed values. Theories for FRB radiation need to address whether the models can reproduce the independence between the distributions of circular polarization degree and burst fluence. Another interesting constraint comes from the nearly constant degree of circular polarization, as observed in Burst 521, which requires that the line of sight should maintain a nearly constant angle relative to the center of the radiation beam in the entire burst. This is possible for the ICS mechanism that predicts a similar $\Pi _{\rm v}$ in a wide range of azimuthal angles with respect to a bunch [11] and may suggest a non-steady plasma flow along the magnetic field lines [9] or a very special geometric configuration of the magnetic field, electron velocity and line of sight for the curvature radiation model.

Our results may also be explained by wave propagation within the magnetosphere. The differences between Bursts 298, 299 and 300 could be due to the three bursts originating from different depths in a magnetosphere, and propagating different distances before escaping. Since circular polarization may be generated during wave propagation, Burst 300 with the least amount of circular polarization might be least affected by propagation effects among the three bursts. Another hint of magnetospheric propagation is that Burst 738 shows a sudden 90$^\circ$ jump in the linear polarization position angle (at $\sim\! 30$ ms in Fig. 1). Similar features have been widely detected in pulsars, mostly in the averaged pulse profiles [45], but sometimes in single pulses as well [46]. Such orthogonal jumps can be attributed to the superposition of two linearly polarized wave modes (O mode and X mode) either incoherently [45] or coherently [47]. For our case, the observed PA jump occurs within a single burst, and the degree of circular polarization forms a peak at the PA-jump epoch accompanied by a nearly full depolarization of the linear polarization. These behaviors suggest a possible coherent superposition between the two linear polarization modes. It is more plausible that the two modes were excited by the same group of electrons, and the propagation effects, such as birefringence, within the magnetosphere induced the 90$^\circ$ PA jump. Our results indicate that the propagation effects also need to maintain a 90$^\circ$ phase difference between the two modes to ensure that linear polarization is depolarized and that circular polarization dominates at the epoch of the PA jump. The multi-path propagation effect [48] can cancel linear polarization and keep circular polarization components, but it is unexpected that the $\Pi _{\rm v}$ peaks at the PA jump.

**Figure 2.**
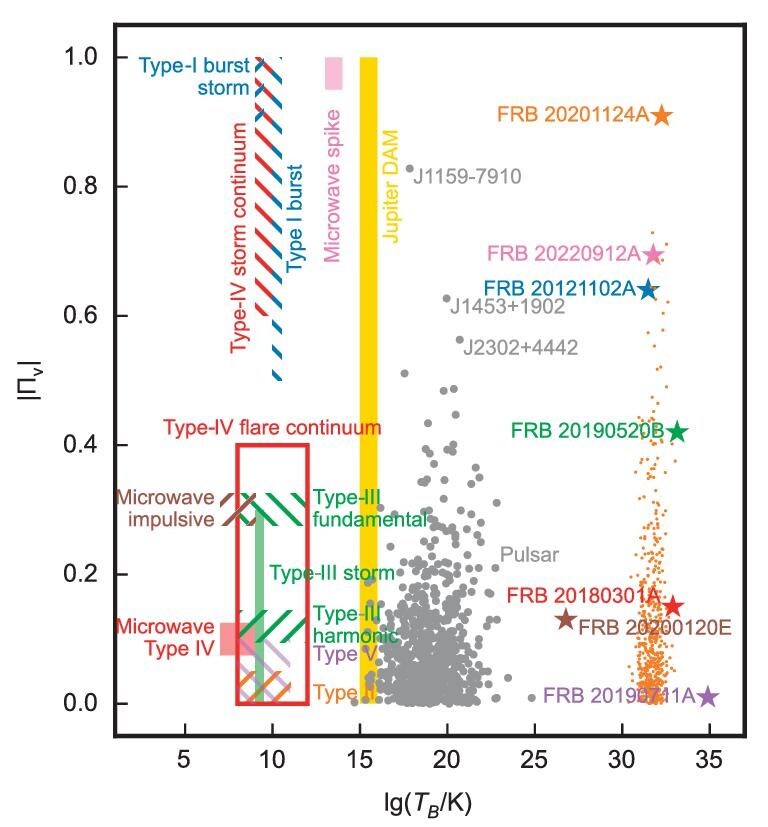
Astrophysical sources with significant circular polarization in the radio band. The $x$ axis is the brightness temperature. The $y$ axis is the degree of circular polarization $\Pi _{\rm v}$. For FRBs, the maximal reported $\Pi _{\rm v}$ is plotted with star symbols. All the bursts of FRB 20201124A with ${\rm S/N}\ge 50$ are also included (orange dots) for the reader’s reference. The details of the data sources are presented in Section S5.

FRB 20201124A belongs to a unique type of source. In Fig. 2, we summarize astrophysical radio sources known so far to exhibit significant circular polarization. One can see that FRB 20201124A stands out in the parameter space of high $\Pi _{\rm v}$ and high luminosity. The other examples with a high degree of circular polarization include Type-I and Type-IV solar storms, as well as solar storm continua and microwave spikes, which can reach a degree of circular polarization similar to that of Burst 521. Additionally, the magnetosphere of Jupiter has been observed to generate highly circularly polarized radio emissions. Unlike in the case of FRBs, these sources of high circular polarization are powered by sub-relativistic plasma. The circular polarization is mainly generated from cyclotron radiation. In contrast, the high luminosity and high degree of circular polarization in the case of FRBs demand relativistic plasma emission in a new parameter regime, which differs from all radio sources known in the past, suggesting extreme conditions for FRB generation and propagation.

In summary, the extreme and diverse polarization properties of FRB 20201124A provide constraints on the physical mechanisms generating the FRB radiation. Although a comparable degree of circular polarization has been observed in solar and Jovian radio emissions, their non-relativistic nature limits the applicability to FRB models. GRB-like models and out-of-magnetosphere propagation models are disfavored for the following reasons: (1) the models cannot account for rapid polarization changes, as seen in Bursts 298, 299 and 300; (2) the frequency independence of $\Pi _{\rm v}$, as observed in Bursts 123 and 521, cannot be interpreted by only invoking Faraday conversion and synchrotron/cyclotron absorption, and (3) no strong correlation between flux and circular polarization is detected. In contrast, pulsar-like models and magnetospheric propagation models are more plausible, even though some fine-tuning of these models may be necessary. Among the known radio burst/pulse sources, the high degree of circular polarization, combined with the high brightness temperature, makes FRB radiation processes even more mysterious.

## METHODS

FAST intensively monitored FRB 20201124A between 25 September and 17 October 2021 [17,32–34]. The L-band receiver covers radio frequencies between 1 and 1.5 GHz, divided into 4096 channels. The original time resolution is $49.152\,\mu{\rm s/sample}$. We used the software package TransientX [49] to search for bursts in the data, and detected more than 500 bursts with $\mathrm{S/N}> 50$ between 25–28 September 2021. More details about the observations and burst detection can be found in Section S1.

The signal from the noise diode was injected to the receiver as the linearly polarized calibrator. The polarimetric calibration was performed using PSRCHIVE [50]. The rotation measure was then measured by fitting the oscillation of Stokes $Q$ and $U$. In this article we follow the PSR/IEEE convention on the definition of Stokes parameters [51]. Our final results shown above include both the corrections of the feed polarization response and Faraday rotation effect. Further details about the polarimetry can be found in Sections S2 and S3.

## Supplementary Material

nwae293_Supplemental_File

## Data Availability

Raw data are archived and available from the FAST data center (http://fast.bao.ac.cn). Owing to the large data volume, we encourage contacting the corresponding author and FAST data center for data transfer. The directly related data that support the findings of this study are available from PSRPKU (https://psr.pku.edu.cn/index.php/publications/frb20201124a/). The CHIME/FRB Public Database can be found at https://www.chime-frb.ca/repeaters/FRB20201124A.

## CODE AVAILABILITY

The software packages to generate the results of this paper are available from PSRCHIVE (http://psrchive.sourceforge.net), TRANSIENTX (https://github.com/ypmen/TransientX) and BEAR (https://psr.pku.edu.cn/index.php/publications/frb180301/).
