# Supplementary material for: Ninety percent circular polarization detected in a repeating fast radio burst
Source: Natl Sci Rev. 2024 Sep 9;12(2):nwae293. doi: 10.1093/nsr/nwae293 (PMC11884807; doi:10.1093/nsr/nwae293)
Supplement: nwae293_Supplemental_File [file nwae293_supplemental_file.pdf]

Supplementary data for
Ninety percent circular polarization detected in a repeating fast radio burst

1 Radio observations and burst detection

FAST observed FRB 20201124A between September 25 and October 17, 2021[1, 2, 3, 4]. In total, we have 19 hours of observations. Bursts were only detected between September 25 and 28. We pointed the central beam of the 19-beam receiver of FAST[5] to the coordinate provided by EVN, RA = $05^{\rm h}08^{\rm m}03.5077^{\rm s}$, Dec = $+26^{\circ}03'38.504''$[6]. The 19-beam receiver covers the frequency range of 1 – 1.5 GHz. Search-mode filterbank data with four Stokes parameters were recorded for polarimetry studies using the pulsar digital backend[7, 5] in PSRFITS format[8]. The full band, i.e. 1 to 1.5 GHz, was divided into 4096 frequency channels, and we record data at the temporal resolution of 49.152 μs per sample.

We follow the rather standard procedures[9, 10] to search for burst signals using the software package TransientX[11, 12]. The software can be found in the 'Code Availability' section. The dispersion delay in cold plasma of free electrons is,

$$\Delta t = \mathcal{D}\frac{\rm DM}{\nu^2}, \tag{S1}$$

where DM is dispersion measure, ν is frequency, and constant $\mathcal{D} = 4.148808\times10^3\,{\rm MHz^2\,pc^{-1}\,cm^3\,s}$. Pulses were de-dispersed with the daily average DM value published previously[1], which are 412.4, 412.2, 412.5 and 411.6 $\rm pc\,cm^{-3}$ for the 4 days from the 25th to the 28th September, respectively. Then, the burst signals were searched using boxcar matched filters with widths ranging from 0.1 to 100 ms with the signal-to-noise ratio threshold of 7. RFIs were mitigated in the search. The continuous narrow band satellite RFIs are removed according to the RFI list, and a zero-DM matched filter is used to identify

wideband RFIs without dispersion[11]. The searching results were later verified by human inspection so that the collected bursts are with a clear dispersive signature. Once the bursts were detected, we further removed data in the 20 MHz band edges at both lower and upper frequency ends for our later analysis.

2 Polarization calibration

The 19-beam receiver of FAST uses orthogonal dual linear polarization feeds to receive radio signals[5]. In the first and the last minutes of the observation, modulated signals from a noise diode were injected as a 45° linearly polarized calibrator. The period of modulated noise signals was 100.663296 ms and their duty cycle was 50%. We used software package DSPSR[13] to fold the modulated noise signals, and then used software package PSRCHIVE[8, 14] to calibrate polarization data with the single-axial model. After the polarimetric calibration, the systematical error is at the level of 0.5%[15, 9, 10]. In this manuscript, we adopt the PSR/IEEE convention[16] for the definitions of the Stokes parameters. We checked and found that the polarization properties are essentially not affected by the DM values we use. For most bursts, the polarization profiles using daily averaged DM or DM from maximizing the structure show no visual differences. Most of the bursts that show large differences are due to their complex structures in the dynamic spectra, for which DM inference is unstable. In this paper, we show the polarisation measurements with daily averaged DM.

3 Measurement and Correction of Faraday Rotation

Linear polarization is rotated with frequency, when radio waves propagate through a magnetized plasma, The change of the position angle is,

$$\Delta \mathrm{PA} = \mathrm{RM}\, \lambda^2, \tag{S2}$$

where λ is the wavelength of the radio wave. Similar to previous work[10], we used the Bayesian method[17, 9] to fit the rotation of Stokes Q and U as a function of frequencies to derive the rotation measures (RMs) of the bursts. We also cross checked the results with the revisited rotation measure

synthesis method[18]. We had corrected the RM contribution caused by the geomagnetic field and Earth ionosphere using the estimation from the software package ionFR[19]. Linearly polarized intensity L, polarized intensity P and PA were calculated after correcting Faraday rotation to the infinite frequency. The generalized Weisberg correction [20, 3] was applied when calculating L and P.

4 Flux Density and Burst Energy

Our flux calibration for all the bursts is identical with the previous work. For the details of flux, we refer readers to Ref.[21, 10, 3]. The flux densities S were estimated using the radiometer equation,

$$S_\nu = \frac{T_{\rm sys}({\rm S/N})}{G\sqrt{2B\tau}}, \tag{S3}$$

where the system temperature $T_{\rm sys} \simeq 24\,{\rm K}$, gain $G \approx 16\,{\rm K\,Jy^{-1}}$[5], and B and τ are the signal bandwidth and burst duration, respectively. The fluences F are calculated by integrating the flux densities over the pulse duration. We compute the burst fluence using three approaches: 1) we average the detected signal within the 500 MHz observing band and 2) we fit the spectrum of a burst with a Gaussian function, then we estimate the fluence using the fitted Gaussian function. Both methods can be affected by scintillation and the limited observing bandwidth, as the true signal central frequency may lay outside of the observing band. In order to avoid the problem, we also take the third approach by generating a subset of the data with the central frequency inside the observing band. However, the number of bursts in the sample is limited. The distribution of burst fluence is shown in Fig. S2, where we also compare the fluence distributions for the bursts with high and low degrees of circular polarization. We note that the conclusion is not affected by how we compute the fluence.

To check if the fluence distribution of the bursts with high degree circular polarization ($\Pi_{\rm v} > 50\%$) are different comparing to that of the bursts with $\Pi_{\rm v} \leq 50\%$, we perform the standard two-sample Kolmogorov-Smirnov test[22] between the fluence distributions. Here, the null hypothesis claims that the fluence distributions of large and small degrees of circular polarization are identical, while the alternative claims that the fluence distributions are different. We get a p-value of 0.39 for fluence averaged in the

observing band, 0.95 for the the Gaussian fitted spectral peak fluence for all the bursts, and 0.30 for the Gaussian fitted spectral peak fluence for the bursts whose peak frequency falls in the observing band in the Kolmogorov-Smirnov test, which cannot reject the null hypothesis, i.e. we cannot differentiate the fluence distributions of the two samples.

To investigate if the null result is caused by the limited number of samples, we performed simulations to evaluate the statistical power of the Kolmogorov-Smirnov test for the current limited sample of bursts. In our simulation, an fluence factor (≤ 1) is multiplied to the fluence of high $\Pi_{\rm v}$ bursts, and then we perform the Kolmogorov-Smirnov test and compute the p-value. The procedure is carried out for a uniform grid of fluence factor from 0.01 to 1.0. We can thus compute the fluence factors at which the null hypothesis can be rejected for a given confidence level. As shown in Fig. S3, one would claim a 2-sigma detection, i.e. p-value of 0.05, when the energy of high $\Pi_{\rm v}$ burst drop to approximately 65% for fluence averaged in the observing bnad, 35% for the Gaussian fitted fluence peak for all the bursts, and 8% for the Gaussian fitted fluence peak for the bursts whose peak falls in the observing band. In this way, our data set is sensitive to 35% fluence difference between the high and low $\Pi_{\rm v}$ bursts, i.e. we would detect the fluence difference of the two population, if the high $\Pi_{\rm v}$ bursts were 35% weaker. Thus, any theoretical model predicting significant correlation between burst energy and $\Pi_{\rm v}$ would be in tension with our observations.

5 Astrophysical sources with significant circular polarization

In Figure 2, we collected the brightness temperature and degree of circular polarization of various astrophysical sources, including solar radio bursts, Jupiter, radio pulsars, and FRBs. Our data source for solar radio bursts are from Ref.[23]. For radio emission from Jupiter and its magnetosphere, there are three possibilities[24]: i) thermal emission from planetary disks, ii) auroral cyclotron emission below 40 MHz, including decimeter (DAM), hectometer (HOM) and broadband kilometer (bKOM) emissions, iii) synchrotron emission between 30 MHz and 30 GHz. Because we focus on the burst-like sources, we

only include the Jovian DAM emission in Figure 2. The brightness temperature of Jovian DAM can reach $> 10^{15}$ K[25], while the degrees of circular polarization of some bursts approach 100%[26].

A fraction of pulsars exhibit strong circular polarization[27]. We include the polarization observation of normal pulsars[28] and millisecond pulsars[29] in Figure 2. We collected the period, flux and pulse width from the ATNF Pulsar Catalogue[30]. The brightness temperature of a pulsar is estimated[31] using $T_b = SD^2c^2/2kA\nu^2$, where S is the observed radio flux, D is the pulsar distance. A is the effective magnetosphere area and estimated using the pulse timescale, i.e. $A = \pi W^2 c^2$, with W being the pulse width at half of peak. Constants k and c are the Boltzmann constant and light speed, respectively.

For FRBs, non-detection or marginal detection of circular polarization has been previously reported for FRB repeaters, e.g. FRB 20121102A[32, 33, 34, 35], FRB 20180301A[36], 20190711A[37], and 20220912A[38]. In addition to FRB 20201124A, circularly polarized emission has been sporadically detected from a few FRB repeaters. Circular polarization has also been reported in bursts from FRB 20220912A[39, 40] (70%) and FRB 20190520B[41] (42%). Recently, circular polarization of 64% from FRB 20121102A was also reported[42]. In Figure 2, the highest values of published $\Pi_{\rm v}$ are collected. The brightness temperature of FRB is estimated[43] using

$$T_b = \frac{SD_{\rm L}^2}{2\pi k_{\rm B}(1+z)\nu^2\Delta t^2}, \tag{S4}$$

where $D_{\rm L}$ is the luminosity distance derived from the redshift z, and, similar to the pulsar case, the burst time width Δt is used to estimate the size of emission zone[43].

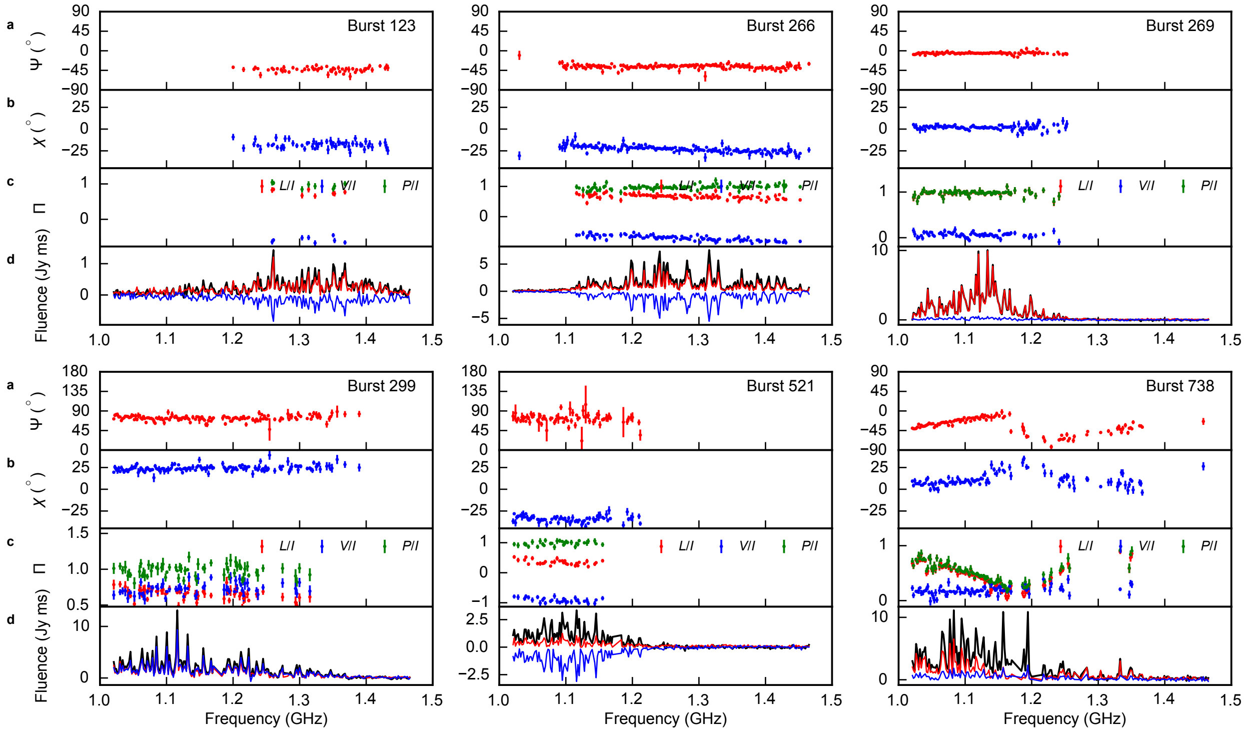

Figure S1: polarization spectra of selected sample of bursts with high degrees of circular polarization or abrupt jumps in linear polarization position angle. For each burst, we plotted a) Position angle, b) Ellipticity angle, c) degree of linear (red), circular (blue) and total polarization (green), and d) Burst fluence of total intensity (black), linearly polarization (red), and circular polarization (blue) as functions of frequency.

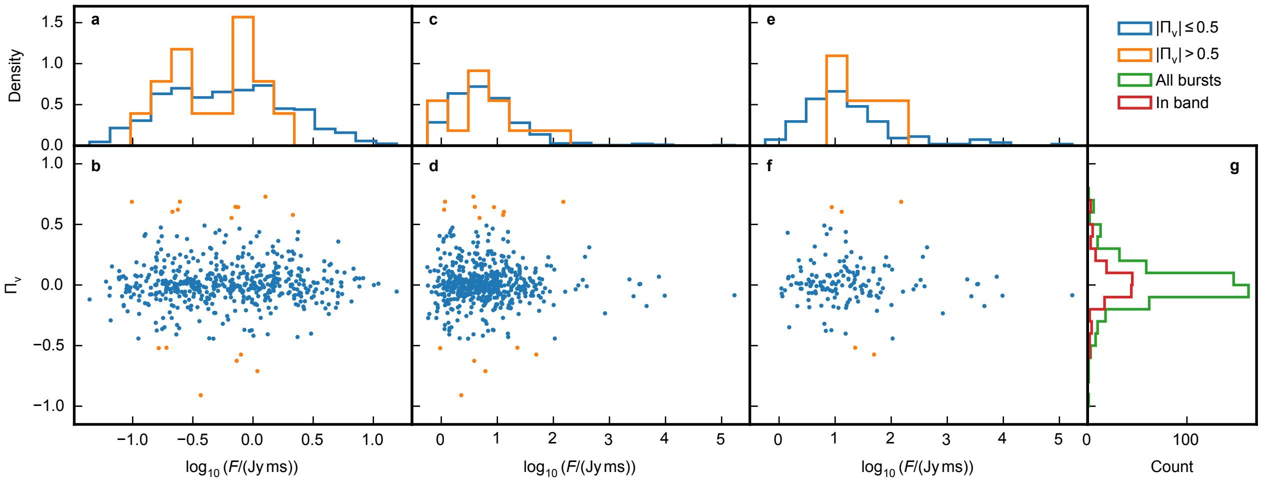

Figure S2: The distribution of burst fluence and degree of circular polarization (Π_v) for bursts with $S/N \geq 50$. a) histogram of burst fluence averaged in the observing band, bursts with $|\Pi_v| \geq 50\%$ are in orange, while the other bursts are in blue. b) two dimensional distribution of burst fluence averaged in the observing band and Π_v with the same color convention of a). c) histogram of burst fluence using Gaussian fitted spectra for all the bursts. d) two dimensional distribution of fluence using Gaussian fitted spectra and Π_v for all the bursts. e) histogram of burst fluence using Gaussian fitted spectra for the bursts with spectral peak in the observing band. f) two dimensional distribution of fluence using Gaussian fitted spectra and Π_v for the bursts with spectral peak in the observing band. g) Histogram of Π_v for all bursts and bursts with spectral peak in the observing band.

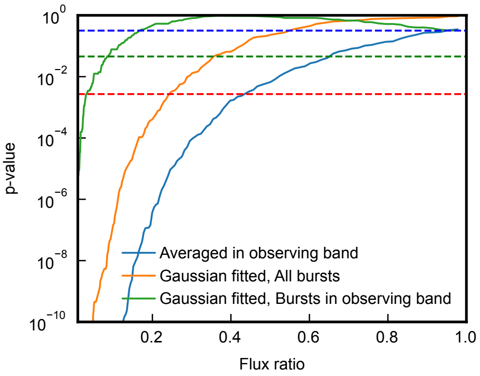

Figure S3: The relation between the p-value of Kolmogorov-Smirnov test and fluence factor multiplied to the fluence of bursts with $|\Pi_v| \geq 50\%$. The blue, orange and green solid curves are the p-value and fluence factor relation for burst fluence averaged in the observing band, Gaussian fitted spectra for all bursts and bursts with spectral peaks in the observing band, respectively. The blue, green, and red dashed lines are for 1-, 2-, and 3-σ, i.e. p-value of 32%, 5%, and 0.3 %, respectively.

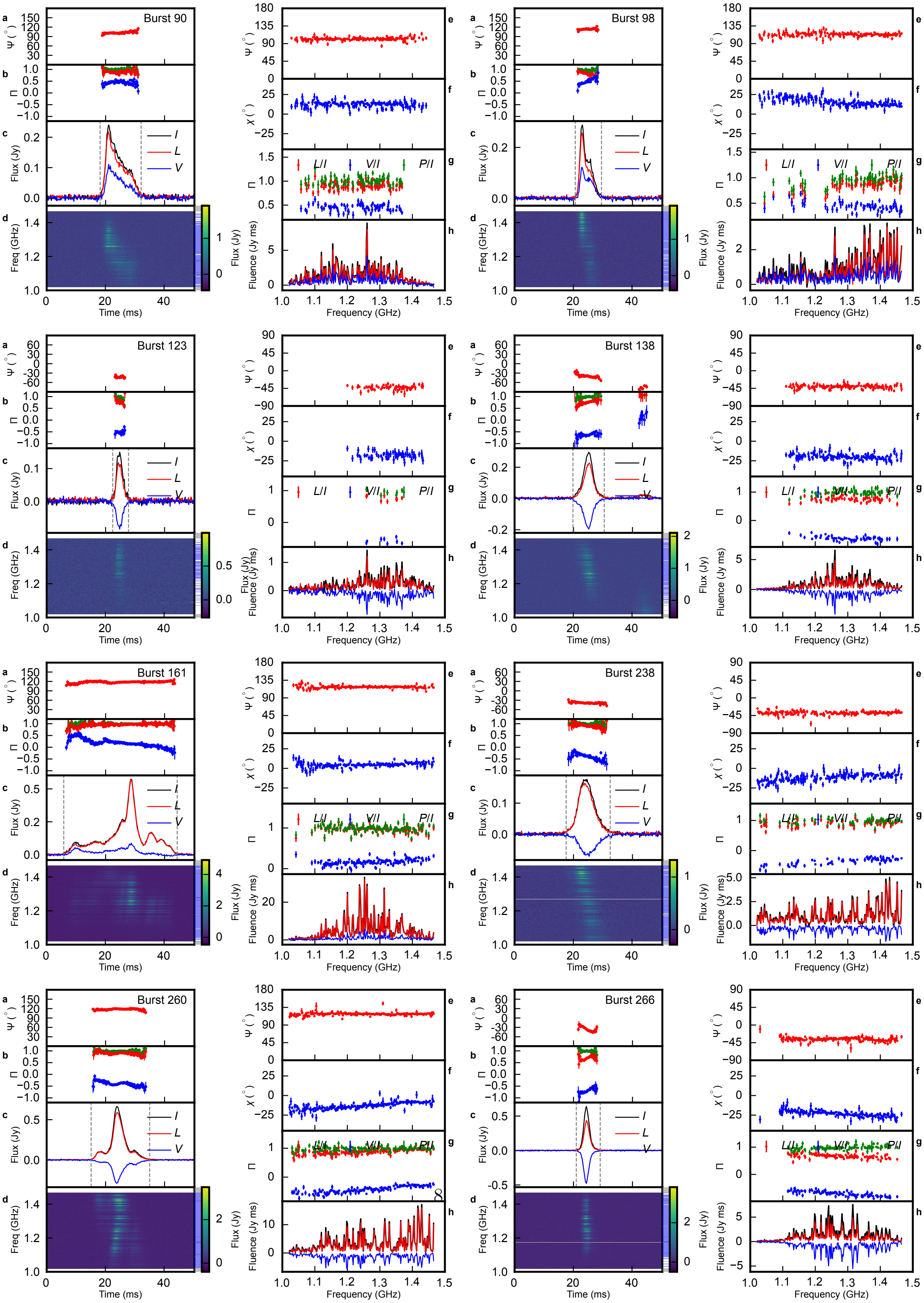

Figure S4: Bursts with $|\Pi_V| \geq 50\%$ and S/N$\geq$ 50. Notations are the same as in Figure 1.

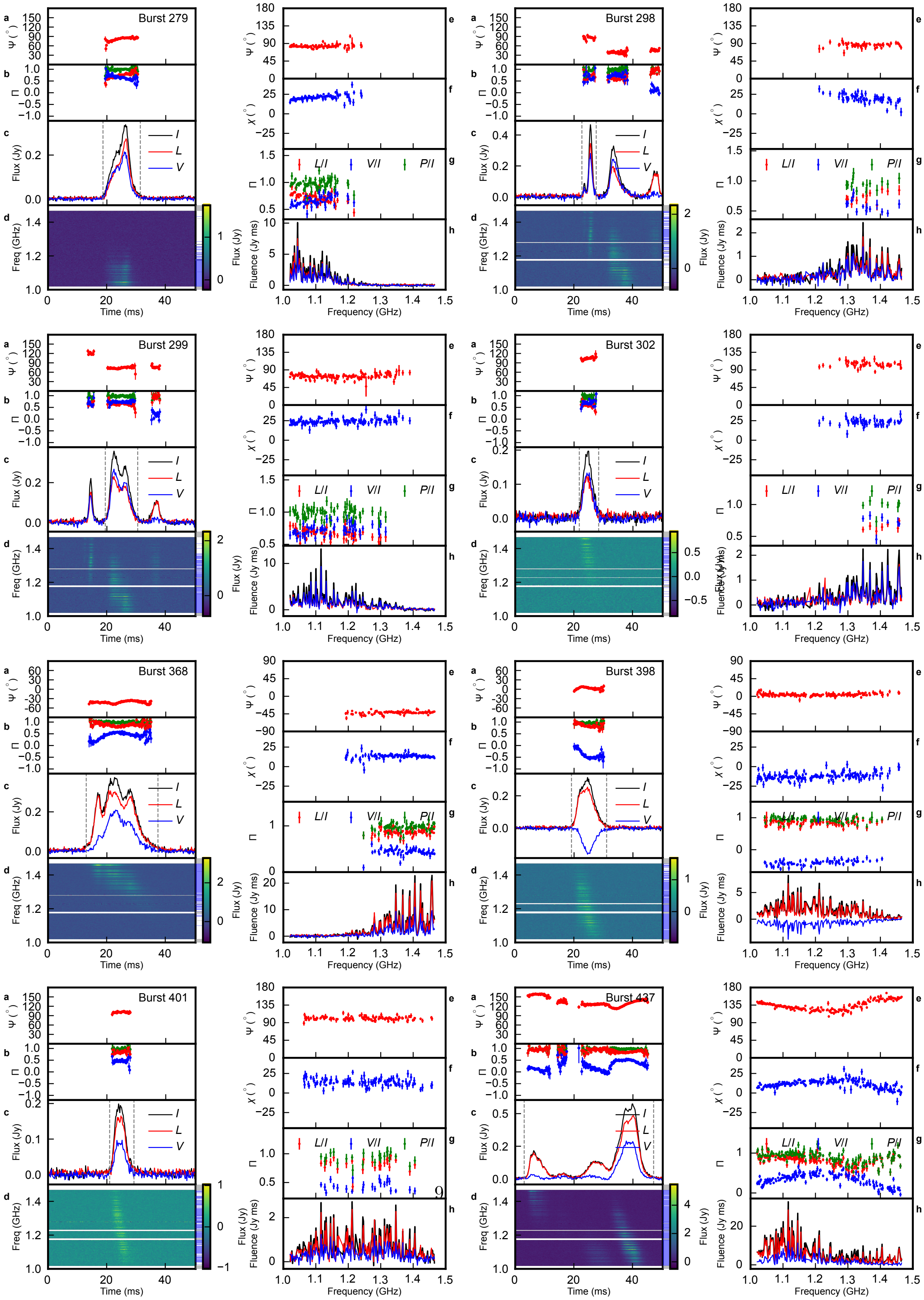

Figure S5: Continuation of Fig. S4.

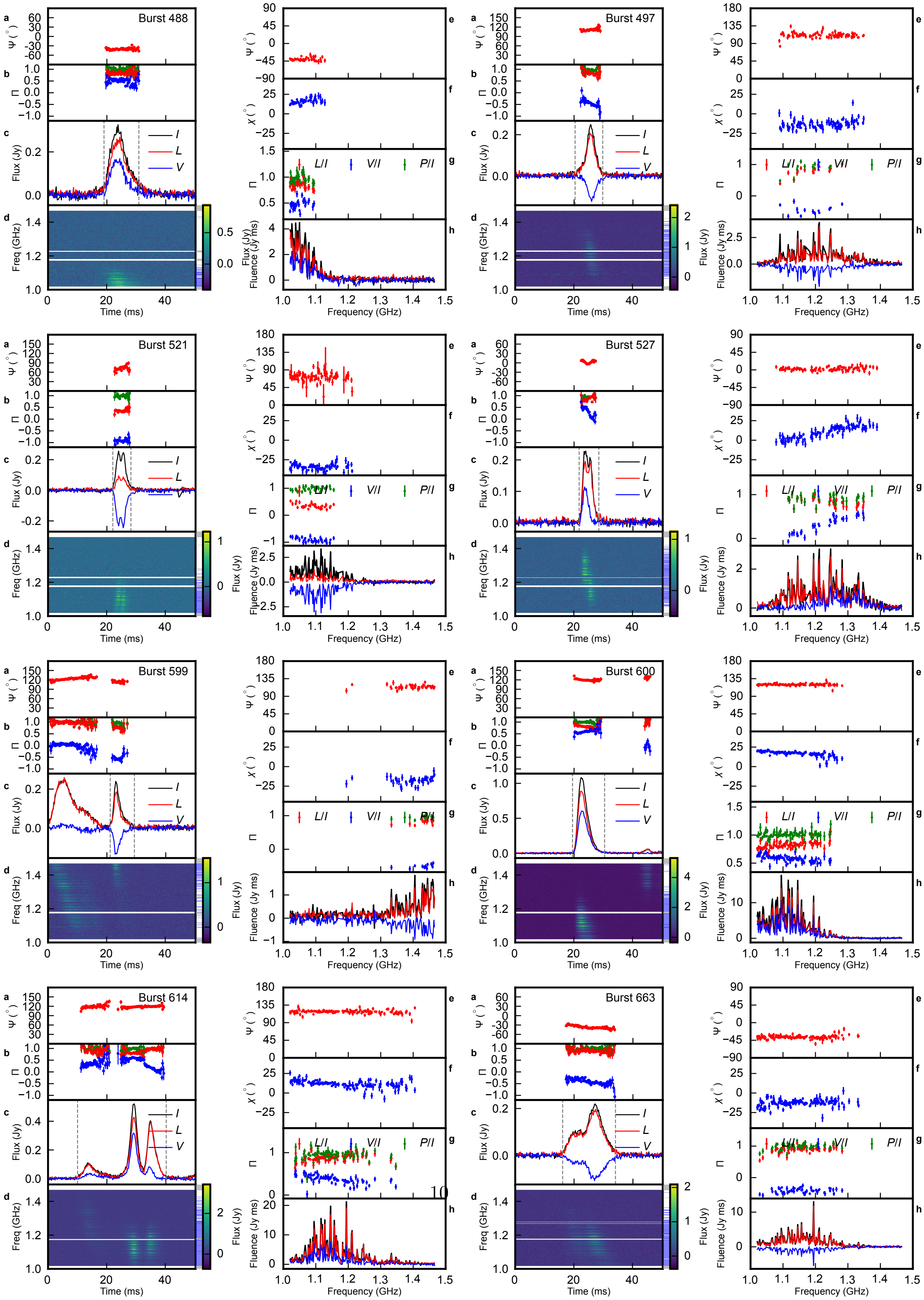

Figure S6: Continuation of Fig. S4.

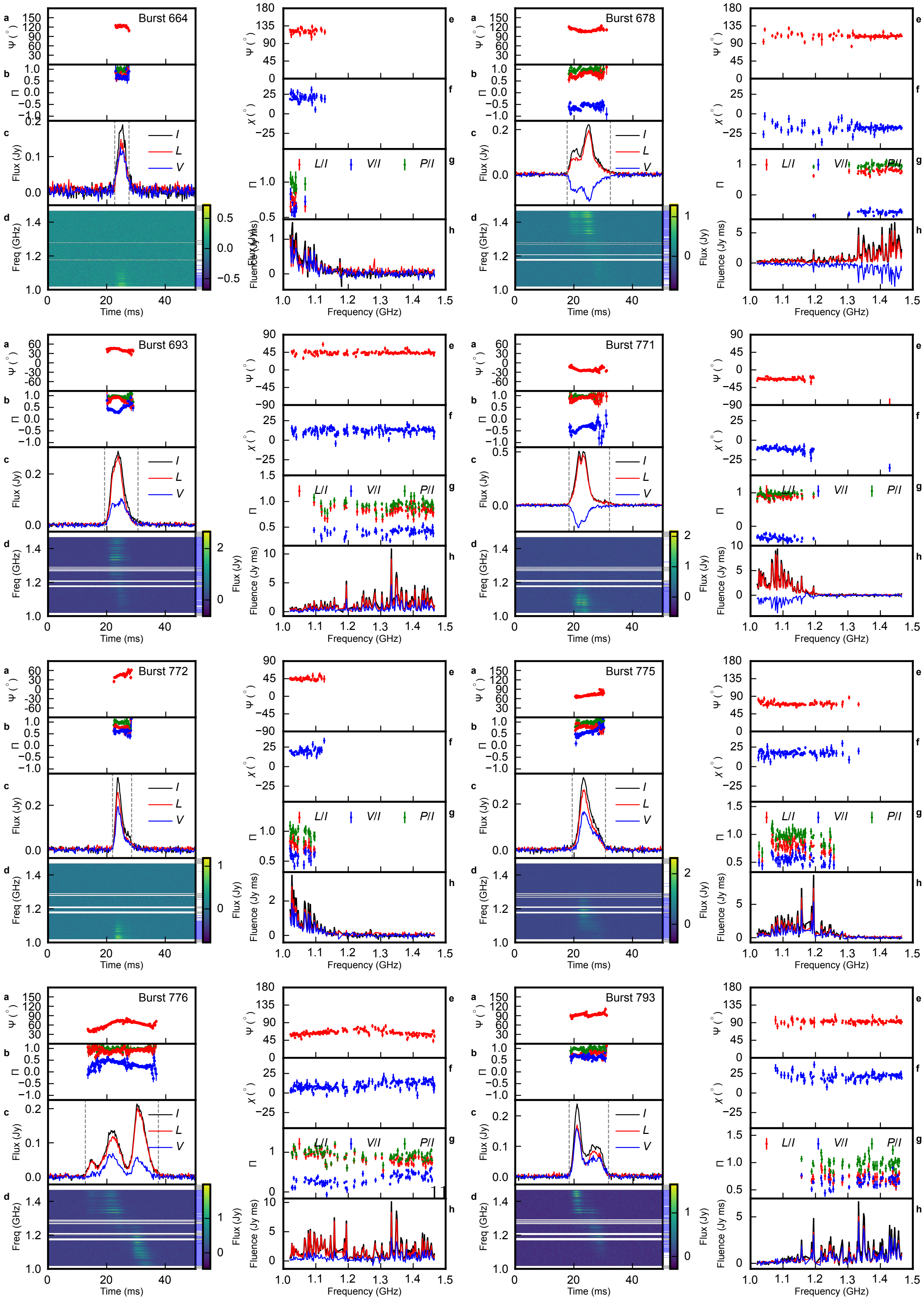

Figure S7: Continuation of Fig. S4.

Table S1: Summary of high Π_v burst properties. Uncertainties are for 68% confidence interval.

No.[a]	TOA[b]	$\mathrm{RM_{Bayes}}$[c]	(L/I)[d]	$(V/I)_{\mathrm{mean}}$[e]	$(V/I)_{\mathrm{peak}}$[f]	P/I[g]	Peak flux	Fluence[h]
	day	$\mathrm{rad\,m^{-2}}$	%	%	%	%	Jy	Jy ms
90	0.818177	-584.9 ± 0.5	88.3 ± 0.7	43.7 ± 0.5	56.5 ± 3.5	98.5 ± 0.7	0.51	1.5
98	0.819599	-591.7 ± 0.5	82.2 ± 0.7	46.5 ± 0.6	61 ± 5	94.4 ± 0.8	0.66	1.0
123	0.825277	-578.9 ± 2.3	75.6 ± 1.7	-52.1 ± 1.6	-57 ± 5	91.8 ± 1.9	0.35	0.4
138	0.828698	-583.5 ± 0.8	75.0 ± 0.7	-62.6 ± 0.6	-68.8 ± 2.3	97.7 ± 0.7	0.69	1.2
161	0.833527	-594.23 ± 0.28	96.34 ± 0.26	16.38 ± 0.19	58 ± 5	97.72 ± 0.26	1.40	5.3
238	0.845965	-590.38 ± 0.35	91.4 ± 0.7	-37.8 ± 0.5	-56 ± 4	99.0 ± 0.7	0.47	1.2
260	0.849867	-593.39 ± 0.2	89.50 ± 0.24	-40.12 ± 0.19	-58.3 ± 3.5	98.08 ± 0.25	1.76	3.5
266	0.851199	-595.6 ± 0.6	66.86 ± 0.35	-71.1 ± 0.4	-80.3 ± 3.5	97.6 ± 0.4	1.70	1.5
279	0.853596	-594.2 ± 0.7	73.0 ± 0.5	64.2 ± 0.5	83 ± 4	97.3 ± 0.6	0.90	1.9
298	1.786479	$-585.3^{+2.7}_{-3.0}$	71.2 ± 1.5	62.1 ± 1.4	72.6 ± 3.2	94.5 ± 1.7	0.73	0.7
299	1.786479	-596.2 ± 0.7	65.6 ± 0.6	72.9 ± 0.6	78.8 ± 2.9	98.0 ± 0.7	0.50	2.1
302	1.786862	-612.6 ± 3.4	61.1 ± 1.6	68.7 ± 1.7	–	91.9 ± 1.8	0.32	0.7
368	1.792096	-622.1 ± 0.7	87.0 ± 0.4	43.79 ± 0.34	59.7 ± 3.3	97.4 ± 0.4	0.66	4.8
398	1.794975	-613.0 ± 0.4	84.3 ± 0.6	-40.7 ± 0.5	-58.1 ± 2.6	93.6 ± 0.7	0.48	1.8
401	1.795033	-621.0 ± 0.8	83.3 ± 1.2	45.2 ± 1.0	54 ± 4	94.8 ± 1.3	0.32	0.9
437	1.797915	-637.9 ± 1.2	79.52 ± 0.32	35.83 ± 0.27	54.9 ± 1.2	87.21 ± 0.33	0.85	6.2
488	1.802788	$-588.4^{+2.1}_{-2.0}$	82.4 ± 1.1	49.0 ± 1.0	59 ± 5	95.9 ± 1.2	3.62	2.0
497	1.803396	-579.7 ± 1.3	81.8 ± 1.3	-43.9 ± 1.1	-55 ± 4	92.9 ± 1.4	0.42	0.9
521	1.804982	$-568.4^{+2.8}_{-2.7}$	34.3 ± 0.9	-90.9 ± 1.1	-100 ± 4	97.2 ± 1.2	0.52	1.1
527	1.805791	-615.4 ± 0.9	84.3 ± 1.1	30.7 ± 0.9	52.4 ± 3.3	89.7 ± 1.1	0.41	0.8
599	1.810067	$-610.8^{+3.3}_{-3.5}$	74.0 ± 1.7	-51.7 ± 1.6	-61 ± 4	90.3 ± 1.9	0.51	0.7
600	1.810074	-613.0 ± 0.4	81.07 ± 0.28	57.80 ± 0.25	69.8 ± 2.4	99.56 ± 0.30	1.90	4.2
614	1.810844	-619.3 ± 0.5	87.7 ± 0.5	37.8 ± 0.4	62.2 ± 1.3	95.5 ± 0.5	0.92	3.6
663	1.814971	-595.3 ± 0.7	88.3 ± 0.7	-41.8 ± 0.6	-55 ± 4	97.7 ± 0.8	0.42	1.9
664	1.815084	$-612.2^{+3.4}_{-3.5}$	74.3 ± 2.2	68.6 ± 2.1	–	101.1 ± 2.5	0.36	0.6
678	1.815578	-614.2 ± 1.0	78.2 ± 0.8	-57.4 ± 0.7	-59.8 ± 3.2	97.0 ± 0.9	0.50	1.3
693	1.817261	-594.9 ± 0.4	83.6 ± 0.6	40.3 ± 0.5	58.4 ± 3.3	92.8 ± 0.6	0.64	1.4
771	1.823548	-597.3 ± 0.8	89.6 ± 0.6	-37.0 ± 0.4	-60.5 ± 3.1	97.0 ± 0.6	1.08	2.5
772	1.823569	$-583.8^{+1.8}_{-1.7}$	75.4 ± 1.2	60.4 ± 1.1	66 ± 4	96.6 ± 1.3	0.70	0.9
775	1.823892	-604.8 ± 0.7	78.4 ± 0.7	55.3 ± 0.6	62 ± 4	96.0 ± 0.7	0.67	1.5
776	1.824116	-613.0 ± 0.5	87.6 ± 0.6	31.7 ± 0.5	54 ± 4	93.2 ± 0.6	0.46	2.0
793	1.824974	-594.8 ± 0.7	71.9 ± 0.7	64.5 ± 0.7	74 ± 4	96.6 ± 0.8	0.60	1.3

[a] Burst index number.
[b] Barycentric burst time of arrival in Barycentric Coordinate Time (TCB) scale. The time reference of $T = 0$ is MJD 59484.
[c] Faraday rotation measure using Bayesian method after correcting the Earth ionosphere contribution.
[d] Average degree of linear polarization.
[e] Average degree of circular polarization.
[f] Peak degree of circular polarization.
[g] Average degree of total polarization.
[h] Average fluence in the burst frequency channels.

References

[1] Zhou DJ, Han JL, Zhang B et al. FAST Observations of an Extremely Active Episode of FRB 20201124A: I. Burst Morphology. Research in Astronomy and Astrophysics 2022; 22:124001.

[2] Zhang YK, Wang P, Feng Y et al. FAST Observations of an Extremely Active Episode of FRB 20201124A. II. Energy Distribution. Research in Astronomy and Astrophysics 2022; 22:124002.

[3] Jiang JC, Wang WY, Xu H et al. FAST Observations of an Extremely Active Episode of FRB 20201124A. III. Polarimetry. Research in Astronomy and Astrophysics 2022; 22:124003.

[4] Niu JR, Zhu WW, Zhang B et al. FAST Observations of an Extremely Active Episode of FRB 20201124A. IV. Spin Period Search. Research in Astronomy and Astrophysics 2022; 22:124004.

[5] Jiang P, Tang NY, Hou LG et al. The fundamental performance of FAST with 19-beam receiver at L band. Research in Astronomy and Astrophysics 2020; 20:064.

[6] Marcote B, Kirsten F, Hessels JWT et al. VLBI localization of FRB 20201124A and absence of persistent emission on milliarcsecond scales. The Astronomer's Telegram 2021; 14603: 1.

[7] Jiang P, Yue Y, Gan H et al. Commissioning progress of the FAST. Science China Physics, Mechanics, and Astronomy 2019; 62:959502.

[8] Hotan AW, van Straten W and Manchester RN. PSRCHIVE and PSRFITS: An Open Approach to Radio Pulsar Data Storage and Analysis. Publications of the Astronomical Society of Australia 2004; 21: 302–309.

[9] Luo R, Wang BJ, Men YP et al. Diverse polarization angle swings from a repeating fast radio burst source. Nature 2020; 586: 693–696.

[10] Xu H, Niu JR, Chen P et al. A fast radio burst source at a complex magnetized site in a barred galaxy. Nature 2022; 609: 685–688.

[11] Men YP, Luo R, Chen MZ et al. Piggyback search for fast radio bursts using Nanshan 26 m and Kunming 40 m radio telescopes - I. Observing and data analysis systems, discovery of a mysterious peryton. Monthly Notices of the Royal Astronomical Society 2019; 488: 3957–3971.

[12] Men Y and Barr E. TransientX: A high-performance single-pulse search package. Astronomy & Astrophysics 2024; 683:A183.

[13] van Straten W and Bailes M. DSPSR: Digital Signal Processing Software for Pulsar Astronomy. Publications of the Astronomical Society of Australia 2011; 28: 1–14.

[14] van Straten W, Demorest P and Oslowski S. Pulsar Data Analysis with PSRCHIVE. Astronomical Research and Technology 2012; 9: 237–256.

[15] Dunning A, Bowen M, Castillo S et al. Design and laboratory testing of the five hundred meter aperture spherical telescope (fast) 19 beam l-band receiver. XXXIInd General Assembly and Scientific Symposium of the International Union of Radio Science (2017) 1–4.

[16] van Straten W, Manchester RN, Johnston S et al. PSRCHIVE and PSRFITS: Definition of the Stokes Parameters and Instrumental Basis Conventions. Publications of the Astronomical Society of Australia 2010; 27: 104–119.

[17] Desvignes G, Kramer M, Lee K et al. Radio emission from a pulsar's magnetic pole revealed by general relativity. Science 2019; 365: 1013–1017.

[18] Schnitzeler DHFM and Lee KJ. Rotation measure synthesis revisited. Monthly Notices of the Royal Astronomical Society 2015; 447: L26–L30.

[19] Sotomayor-Beltran C, Sobey C, Hessels JWT et al. Calibrating high-precision Faraday rotation measurements for LOFAR and the next generation of low-frequency radio telescopes. Astronomy & Astrophysics 2013; 552:A58.

[20] Everett JE and Weisberg JM. Emission Beam Geometry of Selected Pulsars Derived from Average Pulse Polarization Data. Astrophysical Journal Supplement Series 2001; 553: 341–357.

[21] Chime/Frb Collaboration, Amiri M, Andersen BC et al. Periodic activity from a fast radio burst source. Nature 2020; 582: 351–355.

[22] Press W, Teukolsky S, Vetterling WT et al. Numerical Recipes 3rd Edition: The Art of Scientific Computing (Cambridge University Press, New York, NY, USA), 3 edition.

[23] Dulk GA. Radio emission from the sun and stars. Annual Review of Astronomy and Astrophysics 1985; 23: 169–224.

[24] Girard JN, Zarka P, Tasse C et al. Imaging Jupiter's radiation belts down to 127 MHz with LOFAR. Astronomy & Astrophysics 2016; 587:A3.

[25] Clarke TE, Higgins CA, Skarda J et al. Probing Jovian decametric emission with the long wavelength array station 1. Journal of Geophysical Research (Space Physics) 2014; 119: 9508–9526.

[26] Marques MS, Zarka P, Echer E et al. Statistical analysis of 26 yr of observations of decametric radio emissions from Jupiter. Astronomy & Astrophysics 2017; 604:A17.

[27] Han JL, Manchester RN, Xu RX et al. Circular polarization in pulsar integrated profiles. Monthly Notices of the Royal Astronomical Society 1998; 300: 373–387.

[28] Johnston S and Kerr M. Polarimetry of 600 pulsars from observations at 1.4 GHz with the Parkes radio telescope. Monthly Notices of the Royal Astronomical Society 2018; 474: 4629–4636.

[29] Spiewak R, Bailes M, Miles MT et al. The MeerTime Pulsar Timing Array: A census of emission properties and timing potential. Publications of the Astronomical Society of Australia 2022; 39:e027.

[30] Manchester RN, Hobbs GB, Teoh A et al. The Australia Telescope National Facility Pulsar Catalogue. Astronomical Journal 2005; 129: 1993–2006.

[31] Lorimer DR and Kramer M. Handbook of Pulsar Astronomy (Cambridge University Press, New York, NY, USA).

[32] Michilli D, Seymour A, Hessels JWT et al. An extreme magneto-ionic environment associated with the fast radio burst source FRB 121102. Nature 2018; 553: 182–185.

[33] Gajjar V, Siemion APV, Price DC et al. Highest Frequency Detection of FRB 121102 at 4-8 GHz Using the Breakthrough Listen Digital Backend at the Green Bank Telescope. Astrophysical Journal Supplement Series 2018; 863:2.

[34] Faber JT, Gajjar V, Siemion APV et al. Re-analysis of Breakthrough Listen Observations of FRB 121102: Polarization Properties of Eight New Spectrally Narrow Bursts. Research Notes of the American Astronomical Society 2021; 5:17.

[35] Hilmarsson GH, Michilli D, Spitler LG et al. Rotation Measure Evolution of the Repeating Fast Radio Burst Source FRB 121102. Astrophysical Journal Letters 2021; 908:L10.

[36] Kumar P, Luo R, Price DC et al. Spectropolarimetric variability in the repeating fast radio burst source FRB 20180301A. Monthly Notices of the Royal Astronomical Society 2023; 526: 3652–3672.

[37] Day CK, Deller AT, Shannon RM et al. High time resolution and polarization properties of ASKAP-localized fast radio bursts. Monthly Notices of the Royal Astronomical Society 2020; 497: 3335–3350.

[38] Hewitt DM, Hessels JWT, Ould-Boukattine OS et al. Dense forests of microshots in bursts from FRB 20220912A. Monthly Notices of the Royal Astronomical Society 2023; 526: 2039–2057.

[39] Zhang YK, Li D, Zhang B et al. FAST Observations of FRB 20220912A: Burst Properties and Polarization Characteristics. Astrophysical Journal Supplement Series 2023; 955:142.

[40] Feng Y, Li D, Zhang YK et al. An extreme active repeating fast radio burst in a clean environment. arXiv e-prints 2023; arXiv:2304.14671.

[41] Anna-Thomas R, Connor L, Dai S et al. Magnetic field reversal in the turbulent environment around a repeating fast radio burst. Science 2023; 380: 599–603.

[42] Feng Y, Zhang YK, Li D et al. Circular polarization in two active repeating fast radio bursts. Science Bulletin 2022; 67: 2398–2401.

[43] Zhang B. The physics of fast radio bursts. Reviews of Modern Physics 2023; 95:035005.
